# Next generation sequencing based identification of disease-associated mutations in Swiss patients with retinal dystrophies

**DOI:** 10.1038/srep28755

**Published:** 2016-06-29

**Authors:** Amit Tiwari, Angela Bahr, Luzy Bähr, Johannes Fleischhauer, Martin S. Zinkernagel, Niklas Winkler, Daniel Barthelmes, Lieselotte Berger, Christina Gerth-Kahlert, John Neidhardt, Wolfgang Berger

**Affiliations:** 1 Institute of Medical Molecular Genetics, University of Zürich, Wagistrasse 12, CH-8952, Schlieren, Switzerland; 2 Department of Ophthalmology, University Hospital Zürich and University of Zürich, Zürich, Switzerland; 3 Department of Ophthalmology, Inselspital, Bern University Hospital, University of Bern, Switzerland; 4 Zurich Center for Integrative Human Physiology (ZIHP), University of Zürich, Zürich, Switzerland; 5 Neuroscience Center Zurich (ZNZ), University and ETH Zürich, Zürich, Switzerland

## Abstract

Inherited monogenic diseases of the retina and vitreous affect approximately 1 in 2000 individuals. They are characterized by tremendous genetic heterogeneity and clinical variability involving mutations in approximately 250 genes and more than 20 different clinical phenotypes. Clinical manifestations of retinal dystrophies (RDs) range from mild retinal dysfunctions to severe congenital forms of blindness. A detailed clinical diagnosis and the identification of causative mutations are crucial for genetic counseling of affected patients and their families, for understanding genotype-phenotype correlations and developing therapeutic approaches. Using whole exome sequencing (WES) we have established a reliable and efficient high-throughput analysis pipeline to identify disease-causing mutations. Our data indicate that this approach enables us to genetically diagnose approximately 64% of the patients (n = 58) with variant(s) in known disease-associated genes. We report 20 novel and 26 recurrent variants in genes associated with RDs. We also identified a novel phenotype for mutations in *C2orf71* and provide functional evidence for exon skipping due to a splice-site variant identified in *FLVCR1*. In conclusion, WES can rapidly identify variants in various families affected with different forms of RDs. Our study also expands the clinical and allelic spectrum of genes associated with RDs in the Swiss population.

Monogenic retinal dystrophies (RDs) account for vision loss in more than 2 million people worldwide^1^. The genetic basis of RDs is extremely heterogeneous. Mutations in approximately 250 genes are currently known to be responsible for over 20 different clinically distinguished phenotypes. Clinical manifestations for these disorders range from mild dysfunctions (such as night blindness) to severe and early onset RDs (such as Leber congenital amaurosis, LCA). Retinitis pigmentosa (RP) is the most common form of familial RDs affecting 1 in 3500–4000 people. It affects predominantly the rod photoreceptors and can be transmitted in an autosomal recessive (arRP), dominant (adRP) or an X-linked mode of inheritance (XlRP)^1^,^2^. arRP is considered more severe in terms of a younger age of onset and faster disease progression and accounts for over 50–60% of all RP cases. adRP usually is less severe, accounts for 30–40% of RP cases and is associated with a later age of onset^1^,^2^. In addition, incomplete penetrance has been described for a number of dominant gene mutations^3^,^4^,^5^,^6^. XlRP is seen in 5–15% of RP cases^2^. Clinical manifestations in RP typically begin with night blindness. Later, during disease progression, tunnel vision is characteristic and the disease may in some cases lead to complete blindness. Cone dystrophies (COD) and cone-rod dystrophies (CORD) are more severe compared to rod-dominated phenotypes^1^. The age of onset is usually during early adult life. So far there are 33 genes known to be involved in COD or CORD (RetNet: https://sph.uth.edu/retnet/home.htm). Mutations in the respective genes are autosomal dominant or recessive as well as X-linked. LCA is one of the most severe forms of RDs involving rod and cone photoreceptor cells. Patients usually present at birth with severe vision loss and nystagmus^1^. Currently, mutations in 23 genes are known to cause LCA (RetNet). The frequency of the disease is about 1 in 50,000. Stargardt disease (STGD) is one of the most frequent autosomal recessive macular dystrophy in childhood or adolescence with an estimated incidence of 1 in 10,000. Mutations in 4 genes have been reported to cause STGD, with mutations in *ABCA4* gene being the most frequent. Best macular dystrophy (Best MD) is a stationary or slowly progressing form of macular dystrophy with a variable age of onset between childhood and late teenage years. Best MD (due to mutations in *BEST1*) is most commonly inherited in an autosomal dominant manner but autosomal recessive forms have also been reported^7^. In addition, RDs can be either non-syndromic or syndromic (in combination with non-ocular symptoms). Some of the common syndromic forms are characterized by hearing loss (e.g., Usher syndrome), polydactyly (e.g., Bardet-Biedl syndrome, BBS), or renal abnormalities (e.g., Senior–Løken syndrome).

The identification of disease-causing mutations has been tremendously accelerated by implementation of next generation sequencing (NGS) technologies in diagnostic genetic testing in familial cases of RDs but also in sporadic patients. In this study, we employed whole exome sequencing (WES) in 58 patients with 11 clinically different RDs. We were able to identify putative disease causing variants and thus provide a genetic diagnosis in 64% of the cases. The identified mutation spectrum included novel variants and previously described recurrent mutations. Cases and families, where underlying genetic mutations were not identified, might be instrumental for the identification of novel disease-associated genes.

## Results

### Patients

The majority of analyzed patients (n = 58) originated from Switzerland. We cannot however exclude that some patients have migrated to Switzerland (24.3% foreigners in Switzerland; Source: http://www.bfs.admin.ch/). All of the patients were clinically well-defined and included RP (n = 31), STGD (n = 5), LCA (n = 5), CORD (n = 3), RD not classified further (n = 3), COD (n = 3), Macular dystrophy (n = 3), Best disease (n = 2), Choroideremia (n = 1), BBS (n = 1) and Usher syndrome (n = 1). 38 of these were individual cases with no samples from additional family members. For the remaining 20 cases, samples from additional family members were available to perform segregation analysis.

### Whole exome sequencing

**Figure 1:**
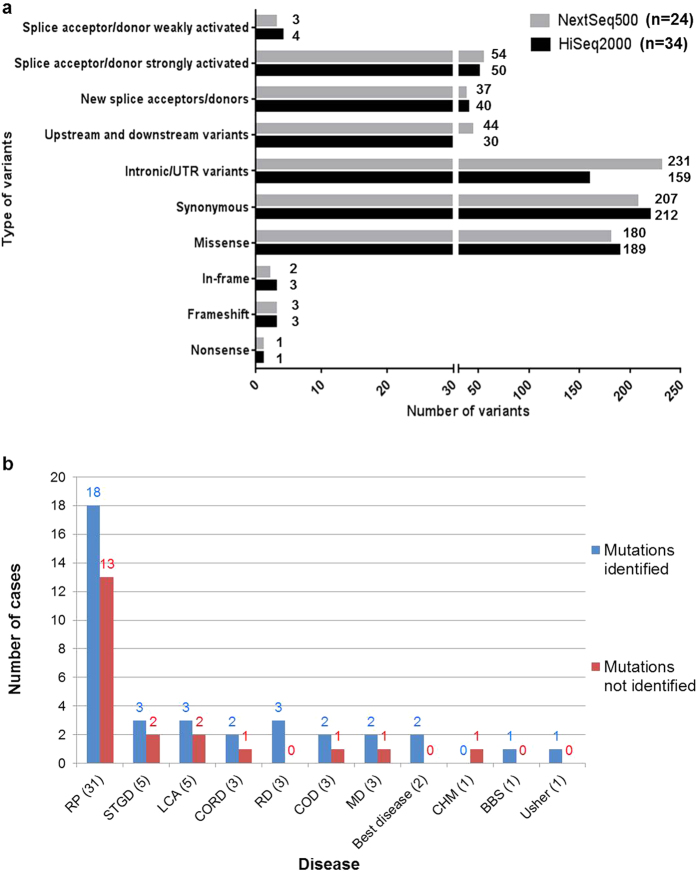
(**a**) Comparison of the numbers and types of variants obtained within 252 genes of interest upon sequencing with Illumina NextSeq500 vs HiSeq2000 platforms. (**b**) Comparison of different disease phenotypes and detection rates of pathogenic mutations.

HiSeq2000 data: 34 cases were sequenced on the HiSeq2000. 7.2–9.3 Gb of data were obtained upon WES that constituted about 9.0*10^7^ reads, with >30 coverage ranging from 81–92% within the different sequences. 550–600 variants were obtained within the known RD genes (n = 252) and an average of approximately 40,000 variants were obtained with WES. Most of the variants identified were synonymous, closely followed by missense variants. A large percentage of the variants lie within the captured intronic regions flanking the exons (Fig. 1a, Supplementary Figure 1). A large number of variants obtained were predicted to affect splicing by either strongly or weakly activating splice acceptor or donor sites.

NextSeq500 data: 24 cases were sequenced on the NextSeq500. Approximately 12 Gb of data were obtained upon WES and 7–9*10^7^ reads, with >20 coverage ranging from 88–93% within the different sequences. The type of variants obtained on the NextSeq500 showed a similar trend as obtained on the HiSeq2000. However, the number of variants obtained was higher with NextSeq500 compared to that of HiSeq2000.

Figure 1a compares the types and numbers of variants obtained within the 252 genes upon sequencing with NextSeq500 versus HiSeq2000. Supplementary Figure 1 compares the type and number of variants obtained within the entire exome data.

### Sequence variants identified in RD cases

**Figure 2:**
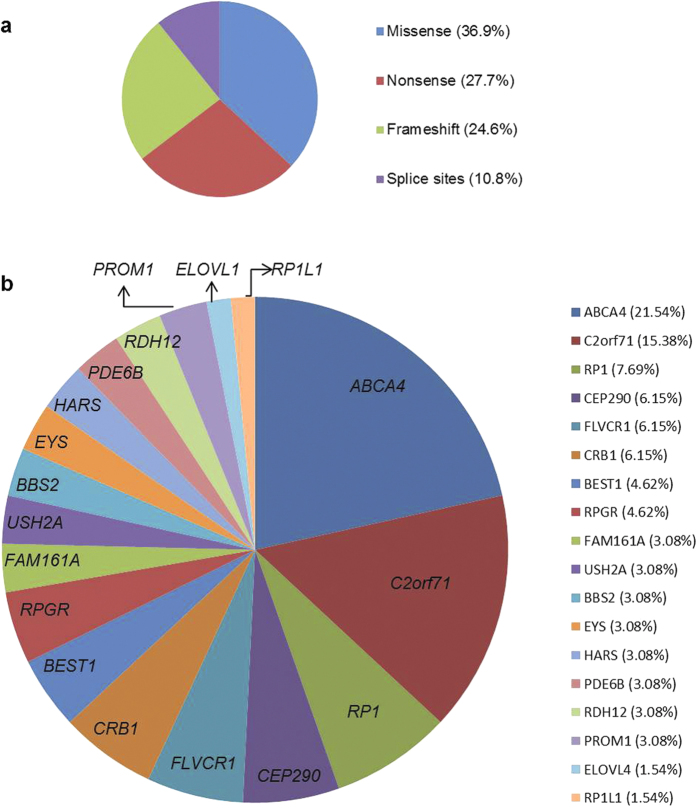
Types and frequencies of sequence variants. (**a**) Distribution of disease-causing variant types within our cohort. (**b**) Frequencies of gene mutations identified within our cohort in cases where a likely genetic diagnosis could be provided.

We performed WES in 58 cases and identified potentially disease-causing variants in known RD genes in 37 (64%) index patients, 25 of whom were simplex/sporadic cases. For 13 cases we had samples from additional family members. Figure 1b shows a comparison of the mutation detection rates in various disease phenotypes. A total of 65 mutant alleles were identified in known RD genes. While 37 (57%) of these have been previously associated with RD, 28 (43%) are novel variants in known RD genes. Amongst the identified disease-associated variants, 24 (36.9%) are missense variants, 18 (27.7%) are nonsense variants, 16 (24.6%) are frameshift deletions or insertions and 7 (10.8%) are predicted to affect splicing (Fig. 2a). In our cohort, the majority of variants were identified in *ABCA4* (21.54%), followed by *C2orf71* (15.38%) and *RP1* (7.69%) and then *CEP290*, *FLVCR1* and *CRB1* each contributing 6.15% to the total variants. Variants were also identified in 12 additional genes (Fig. 2b)

#### ABCA4

**Figure 3:**
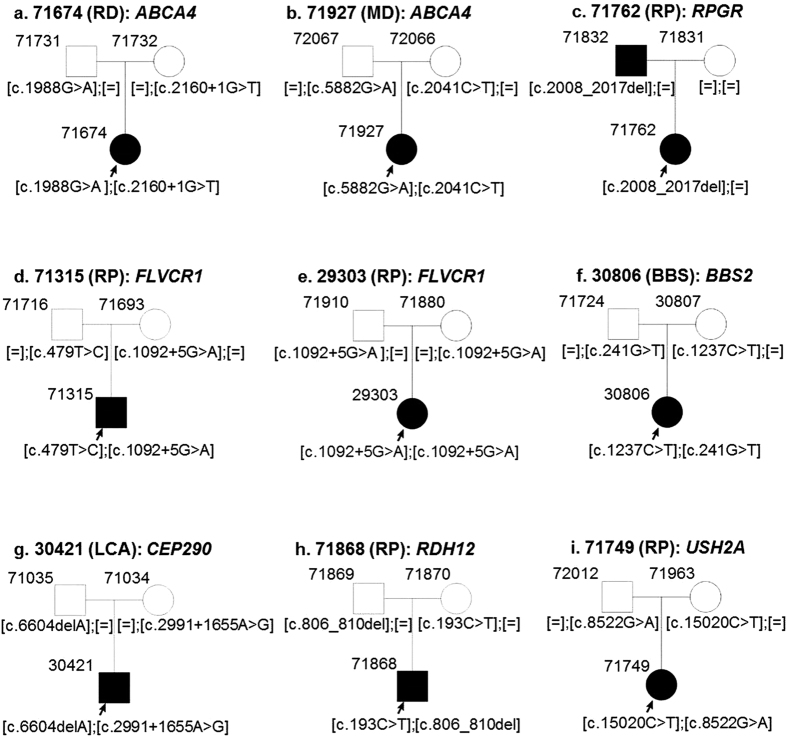
Segregation analysis of disease-causing variants. Index patients are marked with an arrow. All affected individuals are indicated with filled squares or circles.

Mutations in *ABCA4* have been previously associated with STGD, RP, MD, COD or CORD phenotypes^1^,^8^,^9^. In our patient cohort, the majority of variants were identified in *ABCA4* (21.54%) (Fig. 2b), which included 1 RP, 1 RD, 1 MD, 2 COD and 2 STGD cases (n = 7) (Table 1, Fig. 3a,b). We identified 14 mutant alleles in *ABCA4*, including 2 novel variants. In case 71674, diagnosed with arRD, along with a recurrent nonsense mutation (p.Trp663*, HGMD Accession = CM003370), a novel splice-site variant was identified (c.2160 + 1 G > T) (Fig. 3a). This variant is predicted to lead to skipping of exon 14 in the *ABCA4* transcript. The patient inherited one mutant allele from each parent. A novel missense variant was identified in case 71472 diagnosed with RP (Table 1).

#### C2orf71

**Figure 4:**
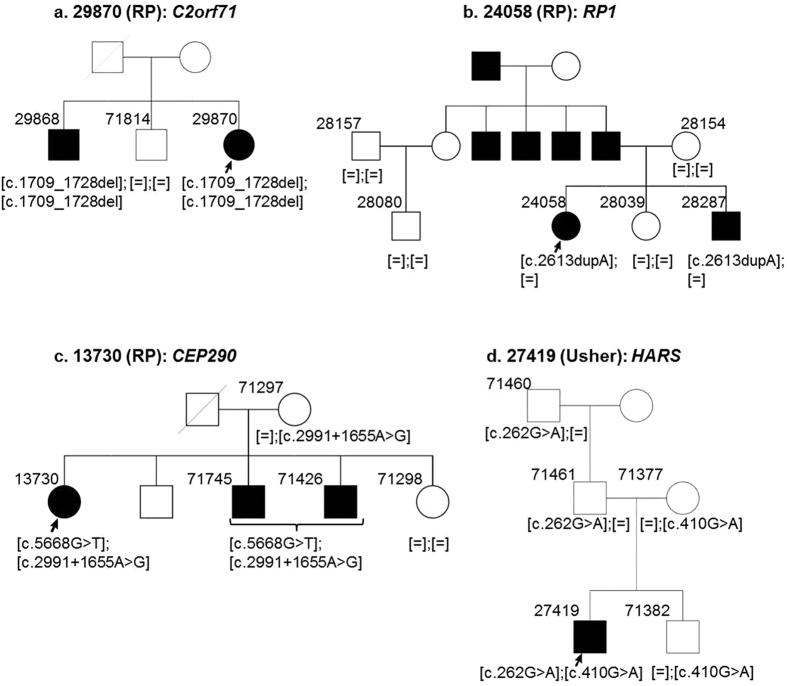
Segregation analysis of disease-causing variants. Index patients are marked with an arrow. All affected individuals are indicated with filled squares or circles.

Mutations in *C2orf71* are known to cause arRP and were first reported by Collen *et al*. in 2010^10^. Within our cohort, mutations in *C2orf71* accounted for the second highest number of mutations (15.38%). We identified potentially pathogenic variants in *C2orf71* in four patients affected with arRP and one diagnosed with CORD (Table 1). Almost 50% of the mutations in HGMD described in *C2orf71* are truncating mutations. Consistently, all the mutations we identified in our patient cohort in *C2orf71* are also truncating mutations including three novel variants (p.Trp650*, p.Gly570Glufs*3, p.Leu744Glufs*7). Index patient 29870 and his affected brother were diagnosed with RP (Fig. 4a) and carried a homozygous frameshift deletion in *C2orf71*: NM_001029883.2: c.1709_1728del:p.Gly570Glufs*3. This mutation was absent in the unaffected brother. Interestingly, we also identified the mutation p.Gly570Glufs*3 in two other cases (71703 and 71688) in a compound heterozygous state with two additional mutations (Table 1). So far mutations in *C2orf71* have been described exclusively for RP. We present a novel phenotype for mutations in this gene, leading to cone-rod dystrophy (Case 71703). The affected sibling of index 71703 was also diagnosed with CORD and carries the same mutations (data not shown). No additional samples from family members were available to perform segregation analysis.

#### RP1

Mutations in *RP1* are known to cause RP and are inherited in autosomal recessive or dominant manner. We identified 2 previously described mutations in 4 patients diagnosed with RP (Table 1): a homozygous nonsense mutation (p.Ser542*, HGMD Accession = CM1211361) in an arRP case; and a heterozygous duplication leading to a frameshift (p.Arg872Thrfs*2, HGMD Accession = CI004598), which was identified in three patients diagnosed with adRP (Table 1). Index patient 24058 has one affected brother and one unaffected sister. They inherited the mutation from their father who had 3 affected brothers and an affected father (Fig. 4b). We also identified this mutation in three additional families, which were not part of this study (data not shown). The rather high frequency of this mutation in our cohort suggests it to be more abundant in the Swiss population. The mutation was absent in 528 control alleles.

#### CEP290

We identified compound heterozygous mutations in *CEP290* in two cases. Index patient 30421 was diagnosed with LCA, while his parents were unaffected (Fig. 3g). A known frameshift deletion NM_025114.3:c.6604del:p.Ile2202Leufs* 24 (HGMD Accession = CD072355) was found in 30421. Index patient 13730 was diagnosed with arRP and has two affected and two unaffected siblings (Fig. 4c). Using WES, a previously described nonsense mutation was identified in 13730: NM_025114.3:c.5668G>T:p.G1890* (HGMD Accession = CM061683). In both cases, the mutations were heterozygous and a mutation in the second allele was missing. Since the patients were diagnosed with classical recessive diseases, we wondered if they carried a common deep intronic mutation in *CEP290*. This mutation NM_025114.3:c.2991+1655A>G (HGMD Accession = CS064383) accounts for 21–43% of mutations in LCA patients^11^,^12^. It leads to an activation of a cryptic splice donor site and inserts a cryptic exon in the messenger RNA of *CEP290*^11^,^12^. Upon Sanger sequencing, we were able to confirm this mutation in patients 30421 and 13730. Analyzing the family members, we confirmed that patient 30421 inherited one mutant allele from each parent (Fig. 3g). Patient 13730 and her affected brother also carried both the mutations, while her unaffected mother was a carrier of the intronic mutation c.2991 + 1655 A > G (Fig. 4c). It can be assumed that the affected members inherited the nonsense mutation in *CEP290* from the deceased father, samples from whom were not available.

#### FLVCR1

**Figure 5:**
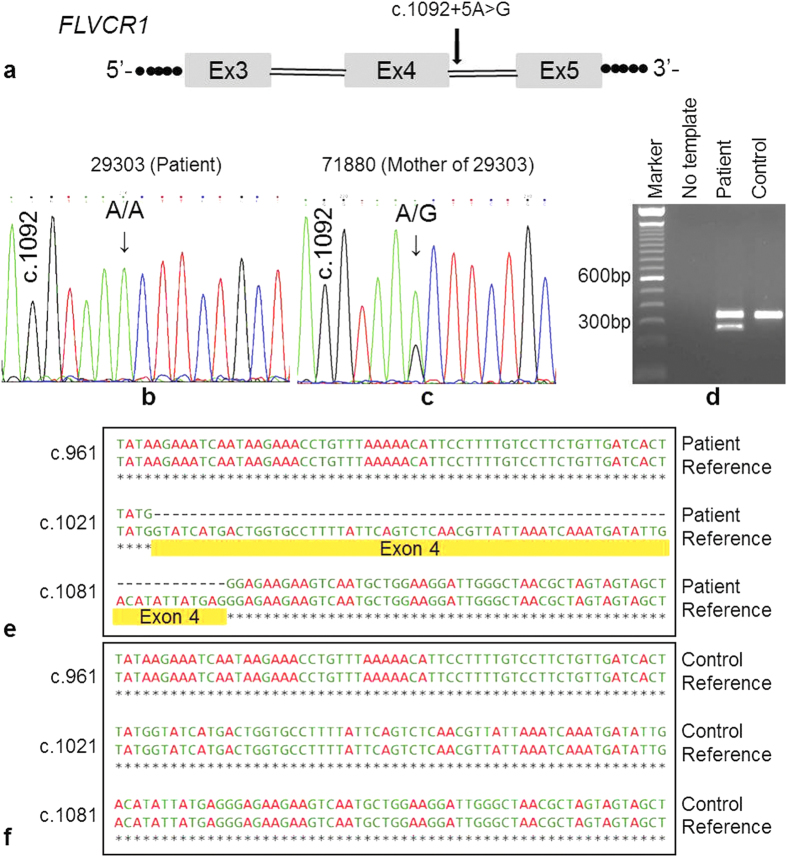
DNA and RNA analysis of the *FLVCR1* mutation c.1092 + 5G > A. (**a**) Partial gene structure of *FLVCR1* is displayed in a schematic drawing. Position of the mutation is indicated with an arrow. This mutation is predicted to affect the canonical splice donor site of exon 4 in *FLVCR1.* (**b**) Sanger sequence of patient DNA showing homozygous mutation c.1092 + 5G > A in *FLVCR1.* (**c**) Sanger sequence of DNA from patient’s mother showing heterozygous mutation c.1092 + 5G > A in *FLVCR1*. Father of the patient was also heterozygous (data not shown). (**d**) Agarose gel image of RT-PCR products from blood RNA. An unrelated, and unaffected individual was used as control. While patient and control both show a product of expected size, the patient sample shows an additional shorter band indicating skipping of exon 4. This was verified by sequencing of both products. (**e**) Alignment of *FLVCR1* cDNA sequence obtained from the patient in comparison to reference showing the skipping of exon 4 (highlighted in yellow). (**f**) Alignment of the *FLVCR1* cDNA sequence from the control sample in comparison to reference showing no skipping of exon 4.

We found a novel missense variant in *FLVCR1* and a previously described splice-site variant in 2 patients diagnosed with RP: c.479 T > C (p.Leu160Pro) and c.1092 + 5 G > A (Table 1). The missense variant affects a highly conserved amino acid and is classified to be disease-causing by 3 different prediction tools (SIFT, PolyPhen2, MutationTaster2). The splice-site variant lies within the consensus splice donor site^13^ and therefore is predicted to affect the normal splicing of exon 4 (Fig. 5a). While case 71315 was compound heterozygous for the above variants, case 29303 is homozygous for the c.1092 + 5 G > A variant. Both patients inherited one mutant allele respectively from each unaffected parent (Figs 3d,e and 5b,c).

### Analysis of *FLVCR1* splicing

The effect of the variant (c.1092 + 5 G > A) on the correct splicing of *FLVCR1* has not been studied previously. We extracted total RNA from venous blood of patient 29303 and performed reverse transcription-PCR (RT-PCR). We analyzed splicing of exons 1 through 7 and detected two isoforms exclusively in the patient: one PCR product of expected size and a second shorter product (Fig. 5d). Upon sequencing of these products, we confirmed that the shorter product lacked exon 4 (Fig. 5e). Exon 4 skipping is highlighted in yellow in the sequence alignment of *FLVCR1* sequences generated from the patient and the reference (Fig. 5e). In contrast, RT-PCR done from control RNA showed normal splicing (Fig. 5d,f). These results clearly show that the variant c.1092 + 5 A > G causes exon skipping from the pre-mRNA by interfering with the correct splicing of *FLVCR1*. Skipping of exon 4 is predicted to introduce a frameshift deletion of 68 base-pairs and consequently a premature termination codon in the mRNA, 31 triplets downstream of Ile343. This truncated mRNA most likely will be targeted for nonsense mediated decay.

#### RPGR

We detected three frameshift deletions in *RPGR* two of which are novel (p.Glu716Glyfs*100 and p.Gln670Argfs*24) (Table 1). The female index patient 71762 and her father were diagnosed with RP, but not her mother (Fig. 3c). The pedigree looked like a classic case of autosomal dominant RP. Sanger screening of the most common adRP associated genes *RHO, PRPF31, RP1* and *PRPH2* did not detect a pathogenic mutation. With WES we observed a novel heterozygous 10 base-pair deletion in *RPGR* (NM_001034853.1:c.2008_2017del:p.Gln670Argfs*24) that leads to a frameshift followed by truncation of the transcript, 24 triplets downstream of the mutation. Mutations in *RPGR* have been associated with complete expression of disease phenotype in hemizygous males and incomplete or variable expression in heterozygous carrier females^14^.

#### HARS

Mutations in this gene have recently been associated with Usher syndrome^15^,^16^. Index patient 27419 was diagnosed with Usher syndrome with no family history for the disease. We identified compound heterozygous missense variants in *HARS*: (i) NM_002109.5:c.262G>A:p.Gly88Ser (Novel) (ii) NM_002109.5:c.410G>A:p.Arg137Gln. This variant has been previously associated with peripheral neuropathy (HGMD accession: CM130192). Both variants substitute highly conserved amino acid residues (from Baker’s yeast to human) and are predicted to be disease-causing by three prediction programs (SIFT, MutationTaster2, Polyphen2). The variants cosegregated in the family (Fig. 4d).

#### Other genes

We found three novel variants in *CRB1* (p.Arg744*, p.Cys183Arg, p.Cys896Ser) in two LCA patients. We also detected recurrent mutations in *BEST1*: homozygous p.Ala135Val and heterozygous p.Ala183Val. Mutations in *BEST1* are known to be inherited in a recessive or dominant fashion. Two novel variants in *USH2A* (p.Pro5007Leu and p.Trp2841*) were also identified in a patient diagnosed with RP. We also observed novel and recurrent variants in *PDE6B, RDH12, EYS, PROM1, ELOVL4* and *RP1L1* (Table 1).

Cases where disease-causing variants were not identified: In 36% of our cases, we were not able to identify the underlying genetic cause of the disease.

## Discussion

We contributed to a recent report describing a panel-based NGS approach of retinal genes^17^. Targeted sequencing of known genes has been successfully employed to identify disease-causing mutations in inherited RDs with variable rates of success between 60% for non-syndromic cases (e.g, RP) and 80% in syndromic cases^17^. Therefore, although very efficient, panel-based sequencing has its limitations. In cases where no disease-associated variants are identified within genes that have been included in the panel, other approaches have to be used to identify the genetic basis of the disease, which adds to costs and time. With 20–40% cases lacking a genetic diagnosis, the costs are significantly higher for a gene diagnostics laboratory and this may in turn lead to significantly higher costs for patients, when genetic testing is not covered by health insurance. In addition, with new genes being rapidly associated to various forms of RDs, modifying the panel becomes expensive. Therefore, we performed WES in our patients, which has the potential of identification of novel disease-associated genes. To our knowledge, this is the first report with WES data generated from human RD cases using NextSeq500. Variant distribution was comparable between both HiSeq2000 and NextSeq500 platforms; however we obtained slightly higher numbers of variants with NextSeq500. A possible reason could be the difference in the chemistries of the two platforms and the different bioinformatic algorithms used to analyze the generated data.

In this study we performed WES in 58 cases diagnosed with various RDs. Most of the patients were of Swiss origin. Wherever possible, samples from additional family members were included to perform segregation analysis. We used a stringent filtering approach that included assessment of multiple parameters such as frequency of the variants, their effect on the encoded protein, and thorough literature search for previously described variants. We identified the underlying genetic basis for the various diseases in 64% of the cases, which is comparable to previously reported panel-based approaches^17^,^18^ and sometimes even higher^19^. In addition to 26 recurrent mutations that have been previously associated with RDs, we detected 20 novel variants that expand the spectrum of mutations causing RDs. Amongst the 20 novel variants identified, 11 are missense variations, while others are loss of function nonsense or frameshift alterations including one variant affecting splicing. Although the bioinformatic algorithms for prediction of missense variants have their limitation, they are accepted as a first attempt to interpret pathogenic relevance of amino acid substitutions. Finally, only functional assays for the respective proteins can provide conclusive evidence. We also present novel phenotypes for known disease-associated genes, thus expanding the clinical spectrum of mutations in these genes, which is of relevance for future gene diagnostics in RD patients.

Within our patient cohort, the majority of variants were identified in *ABCA4,* followed by *C2orf71* and *RP1*. Mutations in *ABCA4* are predominantly known to cause STGD (currently 610 mutations in *ABCA4* have been associated with STGD, source: HGMD), however mutations in *ABCA4* are also associated with RP and CORD^1^,^8^,^9^. Although mutations in *EYS* seem to account for the majority of autosomal recessive RP cases (source: HGMD), in our cohort we identified *EYS* variants in only one case. Similarly, compared to mutations in known disease-associated genes in RD (data not shown; source: HGMD), variants in *USH2A, CRB1* and *PDE6B* were under-represented in our dataset, while mutations in *C2orf71, FLVCR1* and *CEP290* were more frequent in Swiss RD patients. This difference in the frequencies of variants known to be RD-associated could be population-specific, also keeping into account the migration background within the Swiss population.

Since its first association with RP in 2010 by Collin *et al*.^10^, 29 mutations have been identified in *C2orf71* that lead to arRP (source: HGMD). We identified a novel 20 base-pair frameshift deletion in the *C2orf71* gene (NM_001029883.2:c.1709_1728del:p.Gly570Glufs*3) in three of our patients, either in compound heterozygous (71688, 71703) or homozygous state (29870) (Fig. 4a, Table 1). We have identified this mutation in patient 71703 and his affected brother, both of whom were diagnosed with CORD. This presents a novel disease phenotype associated with mutations in *C2orf71*. There is one report which associates mutations in C*2orf71* with CORD type of arRP^20^, but not a true CORD phenotype.

A previous study, to which we contributed, had identified a variant in *FLVCR1* (NM_014053.2:c.1092+5G>A) in a RP patient^17^. Since this variant lies within the highly conserved splice donor consensus sequence at position +5 from the 3’-end of exon 4 in *FLVCR1*, a defect in splicing is very likely. We performed RT-PCR experiments to evaluate the effect of the variant c.1092 + 5 G > A on the splicing of the *FLVCR1* messenger RNA and found that the variant leads to skipping of exon 4 (Fig. 5). In addition, mutations in the *FLVCR1* gene have been associated with posterior column ataxia with retinitis pigmentosa (PCARP). In both our patients described above (71315 and 29303), no posterior column ataxia phenotype was reported until the last clinical examination. *FLVCR1* associated posterior column ataxia has been reported to manifest in the second decade of life^21^. Although the first patient (71315) is rather young (9 years of age), the second patient 29303 (36 years of age) has not shown any signs of neurologic disorders (until the last clinical examination done at the age of 34). It is difficult to comment on whether this might be a novel phenotype associated with mutations in *FLVCR1*. Alternatively, some copies of correctly spliced mRNA could be retained despite the homozygous splice-site variant in patient 29303, which might be sufficient to prevent extraocular neurological symptoms, but only leads to a retinal phenotype. Pathological significance of this exon 4 skipping is currently being studied in our laboratory in more detail. To delineate whether the two patients could be related, we compared informative SNPs which are close to the causative variant in both cases (c.1092 + 5 G > A of *FLVCR1* gene). Since virtually all of the flanking SNPs were heterozygous, even in case 29303 (in whom the *FLVCR1* variant c.1092 + 5 G > A is homozygous), two independent mutation events at the two different alleles are considered more likely. Otherwise, if the mutation would be derived from the same ancestral allele or haplotype, we also should observe homozygosity for SNPs flanking this variant.

In the family of case 71762, an apparent adRP revealed an X-linked inheritance pattern upon WES. The female index patient inherited from her father a pathogenic frameshift deletion mutation in *RPGR* on one allele. X-inactivation of the normal allele could be the most likely possibility for expression of the disease phenotype, as has been reported in earlier studies in case of *RPGR*^14^,^22^.

Using linkage analysis and exome sequencing, Puffenberger *et al*. reported a homozygous mutation in the *HARS* gene in patients of Swiss origin affected with Usher syndrome^15^. Mutations in *HARS* have also been associated with peripheral neuropathies. In case 27419 diagnosed with Usher syndrome, we identified compound heterozygous missense variants in *HARS*. Variant c.262 G > A:p.Gly88Ser was novel while the second mutation c.410 G > A:p.Arg137Gln has been shown to cause loss of function using yeast complementation assays and was associated with peripheral neuropathy^23^. However, this patient has not shown any signs of neurological disorders until the last clinical examination. Upon an initial screening with fewer genes of interest, we had not identified disease associated variants in this case. Only upon the association of the *HARS* gene with Usher syndrome, we searched for variants in Usher syndrome cases in our cohort and identified compound heterozygous pathogenic variants in *HARS* gene in this case. This case highlights the advantage of using WES in comparison with panel-based NGS.

Cases 30421 and 13730 highlight the limitations of WES. In both cases we identified heterozygous pathogenic mutations in *CEP290* on one allele only. The second mutant allele could not be identified by exome capturing methods as it was located in a deep intronic region (c.2991 + 1655 A > G)^11^,^12^. This represents a strong case for complementing exome sequencing with other methods such as whole genome sequencing or Sanger sequencing for specific mutations.

### Detection of multiple disease-associated variants in single patients

Most of our patients carried additional variants in known RD genes that either did not fit the inheritance pattern (e.g. only one recessive variant in an RD-associated gene), or did not pass one of the filtering criteria. The effect of such additional variants is difficult to predict, but these could act as disease modifiers, leading to an earlier or delayed onset/progression of the disease. A list of these variants has been added in supplementary Table 1. These variants were chosen to be pathogenic based on the same criteria that were used to identify disease associated variants.

In conclusion, we have established a highly efficient and reliable whole-exome sequencing and data analysis pipeline that delivers a high diagnostic yield of approximately 65%. In addition, WES provides the advantage that datasets can be reanalyzed as soon as a novel gene for RDs is reported. Wherever possible, we have included additional family members to confirm the diagnosis, an approach which is essential given the fact that patients frequently carry more than one pathogenic mutation in their genome. We have also detected a significant number of novel variants that will aid in further characterizing disease pathogenicity, in addition to identifying novel phenotypes for genes previously associated to some form of RD.

## Materials and Methods

### Patients and families

Patients and family members were referred to us from the eye clinics at the University Hospital Zürich and University Hospital Bern for genetic testing purposes. All patients or family members as well as parents of affected children provided informed consent for genetic testing according to Swiss laws. The clinical diagnosis was established by a comprehensive ophthalmological examination, functional electroretinography (ERG) and morphological studies (optical coherence tomography, OCT; fluorescein angiography, FAG; autofluorescence imaging, AF).

The study was conducted in accordance with the Helsinki Declaration. The approval for genetic testing was awarded to The Institute of Medical Molecular Genetics by the Federal Office of Public Health (FOPH) in Switzerland.

### DNA extraction

Genomic DNA was isolated from patients’ blood samples according to the manufacturer’s recommendations using a coated magnetic bead technology (PerkinElmer Chemagen Technologie GmbH, Baesweiler, Germany). DNA quantification was performed using the Nanodrop (Life technologies, Darmstadt, Germany).

### Whole exome sequencing (WES)

WES was performed either at Atlas Biolabs (Berlin, Germany) using Illumina HiSeq2000 or in-house using Illumina NextSeq500. Sequencing with HiSeq2000 and bioinformatics analysis was done as previously described^24^. For sequencing at the NextSeq500 platform, libraries were generated using Nextera Rapid Capture Exome (Illumina) to generate 150 bp paired-end reads. Alignment of sequence reads, indexing of the reference genome, variant calling and annotation was done with a pipeline based on BWA using BaseSpace Onsite (Illumina). Variants were annotated using Alamut-HT and visualized on Alamut Viewer 2.2 (Interactive Biosoftware, Rouen, France). A filtering pipeline was established to identify the most likely disease-causing variant. Since RDs are rare in the population, only variants with frequency below 1% were chosen. Frequencies of identified variants were also cross-verified on Exome Sequencing Project (ESP) data and Exome Aggregation Consortium (ExAC) data. Mutations that have been previously described to be disease-causing in Human Gene Mutation Database (HGMD) and literature were given the highest priority. The effect of mutations that were described in HGMD were further validated by reading published literature reporting the variants and only those variants that had convincing evidence were chosen. In addition, protein truncation mutations such as nonsense and frameshift (insertions or deletions), were also ranked higher in priority. For variants leading to amino acid substitution, pathogenicity predictions from five programs namely SIFT^25^, MAPP^26^, AGVGD^27^,^28^, MutationTaster2^29^ and Polyphen2^30^ were compared.

To make sure that medically and diagnostically important variants are not being missed, the following approach was used for cases where no disease-associated variants were identified:

- Genes that have been most frequently associated with a certain disease subtype or inheritance pattern were manually screened on Alamut-viewer to check for coverage and variants that could have been missed by the variant caller. For example in patients with Leber congenital amaurosis (LCA) or Usher syndrome, exons of all genes described in Retnet to be associated with LCA or Usher were carefully screened manually.
- If an exon was identified that was not covered and in which known variants have been identified and are included in HGMD, we Sanger sequenced these uncovered exons.
- For recessive cases, if we identified a pathogenic heterozygous variant in one or more of the 252 genes, we screened all exons of these genes. We Sanger sequenced any poorly covered (<10× coverage) or uncovered exons of these genes in order to make sure that a second variant is not being missed.

### RPGR OFR15

*RPGR ORF15* is often poorly covered by NGS capture methods due to its repetitive nature. Patients with *RPGR* mutations show a severe early onset RP phenotype. All male patients diagnosed with an early onset (age <20 years) and severe RP phenotype were screened for *RPGR ORF15* by conventional Sanger sequencing in our laboratory^31^.

### Primer design, PCR amplification and Sanger sequencing

Primer design, PCR and Sanger sequencing was performed as previously described^24^. Sanger sequencing data analysis was performed using the Sequencing Analysis Software v5.4, Sequence Pilot (JSI Medical Systems, Germany) and SeqScape v2.6 (Applied Biosystems, Carlsbad, California, USA) to identify the likely disease causing variants. Mutation is defined as previously described^17^.

## Additional Information

**How to cite this article**: Tiwari, A. *et al*. Next generation sequencing based identification of disease-associated mutations in Swiss patients with retinal dystrophies. *Sci. Rep.* **6**, 28755; doi: 10.1038/srep28755 (2016).

## Supplementary Material

Supplementary Information

## Figures and Tables

**Table 1:** Overview of the 37 cases with clinical diagnosis and most likely disease-causing variants.

S. No.	Case No.	Year of birth	Gender	Clinical Diagnosis	Gene	Mutation (s)	Zygosity	HGMD Accession
1	71134	1980	M	Cone dystrophy	*ABCA4*	NM_000350.2:c.5882G>A:p.Gly1961Glu; NM_000350.2:c.1804C>T:p.Arg602Trp	Compound Heterozygous	CM970016 and CM990025
2	71472	1975	F	Retinitis pigmentosa	*ABCA4*	NM_000350.2:c.4873C>T:p.His1625Tyr	Homozygous	This study
3	71522	1990	M	Cone dystrophy	*ABCA4*	NM_000350.2:c.5882G>A:p.Gly1961Glu; NM_000350.2:c.5461–10T>C	Compound Heterozygous	CM970016 and CS057513
4	71674	2003	F	Retinal dystrophy DD: Retinitis pigmentosa	*ABCA4*	NM_000350.2:c.1988G>A:p.Trp663*; NM_000350.2:c.2160+1G>T	Compound Heterozygous	CM003370 and this study
5	71876	1967	F	Stargardt disease	*ABCA4*	NM_000350.2:c.5381C>A:p.Ala1794Asp; NM_000350.2:c.2401G>A:p.Ala801Thr	Compound Heterozygous	CM990063 and CM070632
6	71927	1989	F	Macular dystrophy	*ABCA4*	NM_000350.2:c.5882G>A:p.Gly1961Glu; NM_000350.2:c.2041C>T:p.Arg681*	Compound Heterozygous	CM970016 and CM990029
7	71882	1989	M	Stargardt disease	*ABCA4*	NM_000350.2:c.6122G>A:p.Gly2041Asp; NM_000350.2:c.5882G>A:p.Gly1961Glu	Compound Heterozygous	CM087709 and CM970016
8	70052	1983	M	Retinitis pigmentosa	*C2orf71*	NM_001029883.1:c.1949G>A:p.Trp650*	Homozygous	This study
9	71688	1967	M	Retinitis pigmentosa	*C2orf71*	NM_001029883.2:c.947del:p.Asn316Metfs*7; NM_001029883.2:c.1709_1728del:p.Gly570Glufs*3	Compound Heterozygous	CD102940 and this study
10	29870	1969	F	Retinitis pigmentosa	*C2orf71*	NM_001029883.2:c.1709_1728del:p.Gly570Glufs*3	Homozygous	This study
11	71703	1991	M	Cone-rod dystrophy	*C2orf71*	NM_001029883.2:c.2227_2228del:p.Leu744Glufs*7; NM_001029883.2:c.1709_1728del:p.Gly570Glufs*3	Compound Heterozygous	This study
12	71918	1983	F	Retinitis pigmentosa	*C2orf71*	NM_001029883.2:c.3002G>A:p.Trp1001*	Homozygous	CM113611
13	71471	2004	F	Retinitis pigmentosa	*RP1*	NM_006269.1:c.1625C>G:p.Ser542*	Homozygous	CM1211361
14	71728	1959	M	Retinitis pigmentosa	*RP1*	NM_006269.1:c.2613dup:p.Arg872Thrfs*2	Heterozygous	CI004598
15	28865	1960	M	Retinitis pigmentosa	*RP1*	NM_006269.1:c.2613dup:p.Arg872Thrfs*2	Heterozygous	CI004598
16	24058	1946	F	Retinitis pigmentosa	RP1	NM_006269.1:c.2613dup:p.Arg872Thrfs*2	Heterozygous	CI004598
17	71192	1977	M	Retinal dystrophy	*RPGR*	NM_001034853.1:c.2143_2144dup:p.Glu716Glyfs*100	Hemizygous	This study
18	71762	2000	F	Retinitis pigmentosa	*RPGR*	NM_001034853.1:c.2008_2017del:p.Gln670Argfs*24	Heterozygous	This study
19	72007	1992	M	Retinitis pigmentosa	*RPGR*	NM_001034853.1:c.2236_2237del:p.Glu746Argfs*23	Hemizygous	CD004113
20	13730	1951	F	Retinitis pigmentosa	*CEP290*	NM_025114.3:c.2991+1655A>G; NM_025114.3:c.5668G>T:p.Gly1890*	Compound Heterozygous	CS064383 and CM061683
21	30421	2008	M	Leber Congenital Amaurosis	*CEP290*	NM_025114.3:c.2991+1655A>G; NM_025114.3:c.6604del:p.Ile2202Leufs*24	Compound Heterozygous	CS064383 and CD072355
22	71315	2006	M	Retinitis pigmentosa	*FLVCR1*	NM_014053.2:c.1092 + 5G>A; NM_014053.2:c.479T>C:p.Leu160Pro	Compound Heterozygous	CS140551 and this study
23	29303	1979	F	Retinitis pigmentosa	*FLVCR1*	NM_014053.2:c.1092 + 5G>A	Homozygous	CS140551
24	71133	2011	F	Leber Congenital Amaurosis	*CRB1*	NM_201253.2:c.2230C>T:p.Arg744*	Homozygous	This study
25	71161	2009	F	Leber Congenital Amaurosis	*CRB1*	NM_201253.2:c.547T>C:p.Cys183Arg; NM_201253.2:c.2687G>C:p.Cys896Ser	Compound Heterozygous	This study
26	70946	1980	F	Best macular dystrophy	*BEST1*	NM_001139443.1:c.404C>T:p.Ala135Val	Homozygous	CM004430
27	70559	1991	M	Best macular dystrophy	*BEST1*	NM_001139443.1:c.548C>T:p.Ala183Val	Heterozygous	CM000841
28	71583	1971	F	Retinal dystrophy	*FAM161A*	NM_001201543.1:c.1807G>T:p.Glu603*	Homozygous	This study
29	71749	1990	F	Retinitis pigmentosa	*USH2A*	NM_206933.2:c.15020C>T:p.Pro5007Leu; NM_206933.2:c.8522G>A:p.Trp2841*	Compound Heterozygous	This study
30	30806	1998	F	Bardet-Biedl Syndrome	*BBS2*	NM_031885.3:c.1237C>T:p.Arg413*; NM_031885.3:c.241G>T:p.Gly81Cys	Compound Heterozygous	CM033336 and CM114523
31	27419	1992	M	Usher syndrome	*HARS*	NM_002109.5:c.410G>A:p.Arg137Gln; NM_002109.5:c.262G>A:p.Gly88Ser	Compound Heterozygous	CM130192 and this study
32	25939	1978	M	Retinitis pigmentosa	PDE6B	NM_000283.3:c.810C>A:p.Cys270*; NM_000283.3:c.811G>A:p.Glu271Lys	Compound Heterozygous	CM962548 and this study
33	71868	2003	M	Retinitis pigmentosa DD: Retinal dystrophy	*RDH12*	NM_152443.2:c.193C>T:p.Arg65*; NM_152443.2:c.806_810del:p.Ala269Glyfs*2	Compound Heterozygous	CM054831 and CD042224
34	71808	1960	M	Retinitis pigmentosa	*EYS*	NM_001292009.1:c.8713T>G:p.Cys2905Gly; NM_001292009.1:c.8269G>C: p.Ala2757Pro	Compound Heterozygous	This study
35	71094	1974	M	Cone-rod dystrophy	*PROM1*	NM_006017.2:c.380G>A:p.Gly127Glu	Homozygous	This study
36	71718	1983	F	Stargardt disease	*ELOVL4*	NM_022726.3:c.810C>G:p.Tyr270*	Heterozygous	CM045143
37	71780	1979	M	Macular dystrophy	*RP1L1*	NM_178857.5:c.1138G>A:p.Gly380Arg	Heterozygous	This study

DD: Differential diagnosis.
