# Supplementary material for: Next generation sequencing based identification of disease-associated mutations in Swiss patients with retinal dystrophies
Source: Sci Rep. 2016 Jun 29;6:28755. doi: 10.1038/srep28755 (PMC4926080; doi:10.1038/srep28755)
Supplement: Supplementary Information [file srep28755-s1.pdf]

Next generation sequencing based identification of disease-associated mutations in Swiss patients with retinal dystrophies

Amit Tiwari[1], Angela Bahr[1], Luzy Baehr[1], Johannes Fleischhauer[3♯], Martin Zinkernagel[2], Niklas Winkler[3], Daniel Barthelmes[3], Lieselotte Berger[2], Christina Gerth-Kahlert[3], John Neidhardt[1+], Wolfgang Berger[1,4,5*]

Affiliations:

1. Institute of Medical Molecular Genetics, University of Zürich, Wagistrasse 12, CH-8952, Schlieren, Switzerland;
2. Department of Ophthalmology, University Hospital Bern, Bern, Switzerland;
3. Department of Ophthalmology, University Hospital Zürich and University of Zürich, Zürich, Switzerland;
4. Zurich Center for Integrative Human Physiology (ZIHP), University of Zürich, Zürich, Switzerland;
5. Neuroscience Center Zurich (ZNZ), University and ETH Zürich, Zürich, Switzerland

+Address since July 1st 2014: Human Genetics, Faculty of Medicine and Health Sciences, University of Oldenburg, 26129 Oldenburg, Germany; Research Center Neurosensory Science, University Oldenburg, 26111 Oldenburg, Germany

♯ Current address: Talacker Augen Zentrum Zürich, Talacker 42, CH-8001 Zürich, Switzerland

*Correspondence to:

Prof. Dr. Wolfgang Berger, Institute of Medical Molecular Genetics, University of Zurich, Wagistrasse 12, CH-8952, Schlieren, Switzerland. Phone: +41 44 556 33 50, Fax: +41 44 556 33 51, Email: berger@medmolgen.uzh.ch

Supplementary Figure 1: Comparison of types and numbers of variants obtained upon WES by Illumina NextSeq500 Vs Illumina HiSeq2000

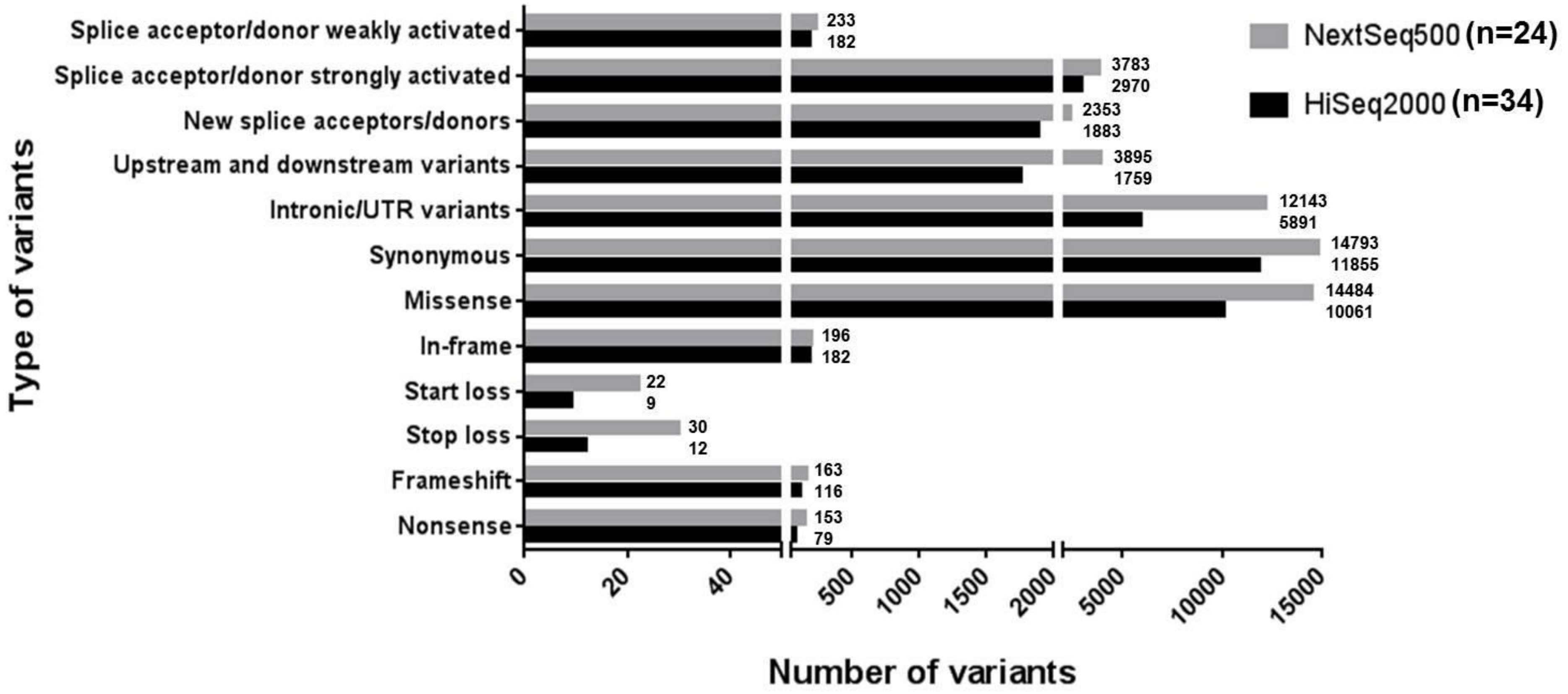

Supplementary Table 1: Additional variants that were deemed non-causal to the phenotype in the patients. These include novel variants of uncertain significance and previously described disease-associated variants that either did not explain the clinical phenotype or the inheritance pattern.

S. No.	Case No.	Clinical Diagnosis	Gene	Variant (s)	SIFT	MAPP	AGVGD Class	PolyPhen2	Mutation Taster2	Predicted effect on splicing	Zygosity	HGMD Accession
1	71134	Cone dystrophy	*VCAN*	NM_004385.4:c.1895C>T:p.Thr632Met	Deleterious	Bad	C15	Benign (0.081)	Polymorphism		Heterozygous	This study
			CDH23	NM_022124.5:c.7748C>T:p.Ala2583Val	Deleterious	Bad	C0	Probably damaging (0.995)	Disease-causing		Heterozygous	This study
			TTLL5	NM_015072.4:c.2950A>T:p.Ser984Cys	Deleterious	Unknown	C0	Probably damaging (1)	Disease-causing		Heterozygous	This study
			CACNA2D4	NM_172364.4:c.1213G>A:p.Asp405Asn	Deleterious	Bad	C15	Probably damaging (1)	Disease-causing		Heterozygous	This study
			CACNA2D4	NM_172364.4:c.2551+9671G>A						New acceptor site	Heterozygous	This study
2	71472	Retinitis pigmentosa	*PEX7*	NM_000288.3:c.377A>C:p.Gln126Pro	Deleterious	Bad	C15	Probably damaging (0.822)	Disease-causing		Heterozygous	This study
			BBS1	NM_024649.4:c.1324C>T:p.Arg442Trp	Deleterious	Good	C0	Probably damaging (1)	Disease-causing		Heterozygous	This study
			RCBTB1	NM_018191.3:c.158G>A:p.Cys53Tyr	Deleterious	Bad	C0	Probably damaging (1)	Disease-causing		Heterozygous	This study
			SLC24A1	NM_004727.2:c.1976C>T:p.Ser659Leu	Deleterious	Good	C0	Possibly damaging (0.626)	Disease-causing		Heterozygous	This study
			DHX38	NM_014003.3:c.901G>A:p.Glu301Lys	Deleterious	Bad	C55	Benign (0.026)	Disease-causing		Heterozygous	This study
3	71522	Cone dystrophy	*KIF7*	NM_198525.2:c.2114A>C:p.Gln705Pro	Deleterious	Bad	C0	Probably damaging (0.984)	Disease-causing		Heterozygous	This study

			PITPNM3	NM_031220.3:c.1656C>G:p.Ile552Met	Deleterious	Bad	C1	Probably damaging (0.973)	Disease-causing		Heterozygous	This study
4	71674	Retinal dystrophy DD: Retinitis pigmentosa	*VPS13B*	NM_017890.4:c.8978A>G:p.Asn2993Ser	Deleterious	Bad	C0	Probably damaging (0.997)	Disease-causing		Heterozygous	CM041280
			PCDH15	NM_001142763.1:c.4329_4337del:p.Pro1446_Pro1448del							Heterozygous	This study
			DHX38	NM_014003.3:c.3662C>T:p.Thr1221Met	Deleterious	Bad	C15	Probably damaging (1)	Disease-causing		Heterozygous	This study
			USH1G	NM_173477.4:c.310A>G:p.Met104Val	Tolerated			Benign (0.081)			Heterozygous	CM149898
5	71876	Stargardt disease	*RHO*	NM_000539.3:c.659T>G:p.Phe220Cys	Deleterious	Bad	C65	Probably damaging (0.991)	Disease-causing		Heterozygous	CM930662
			COL9A1	NM_001851.4:c.1349A>G:p.Glu450Gly	Deleterious	Bad	C66	Probably damaging (1)	Disease-causing		Heterozygous	This study
			CEP290	NM_025114.3:c.4522C>T:p.Arg1508*							Heterozygous	This study
6	71927	Macular dystrophy	*CNGA3*	NM_001298.2:c.1618G>A:p.Val540Ile	Tolerated	Good	C0	Possibly damaging (0.465)	Disease-causing		Heterozygous	CM101951
			RIMS1	NM_014989.5:c.2894C>T:p.Pro965Leu	Deleterious		C25	Possibly damaging (0.713)	Disease-causing		Heterozygous	This study
			CDHR1	NM_033100.3:c.416C>G:p.Pro139Arg	Deleterious		C0	Probably damaging (1)	Disease-causing		Heterozygous	This study
			RPGRIP1	NM_020366.3:c.2066T>C:p.Leu689Pro	Deleterious	Bad	C0	Probably damaging (1)	Polymorphism		Heterozygous	This study
7	71882	Stargardt disease	*FLVCR1*	NM_014053.3:c.661C>T:p.Pro221Ser	Deleterious		C65	Probably damaging (0.999)	Disease-causing		Heterozygous	This study

			MERTK	NM_006343.2:c.791C>G:p.Ala264Gly	Deleterious	Bad	C65	Probably damaging (1)	Disease-causing		Heterozygous	CM1312856
			DNAH5	NM_001369.2:c.12709G>T:p.Val4237Phe	Deleterious	Bad	C0	Benign (0.193)	Disease-causing		Heterozygous	This study
			ADGRV1	NM_032119.3:c.2261T>C:p.Val754Ala	Deleterious	Bad	C25	Possibly damaging (0.682)	Disease-causing		Heterozygous	This study
8	70052	Retinitis pigmentosa	*TMEM237*	NM_001044385.2:c.43-1G>C						Splice acceptor lost	Heterozygous	This study
			NBAS	NM_015909.3:c.2116C>T:p.His706Tyr	Deleterious		C65	Possibly damaging (0.828)	Disease-causing		Heterozygous	This study
9	71688	Retinitis pigmentosa	*RPGRIP1*	NM_020366.3:c.3358A>G:p.Ile1120Val	Tolerated	Good	C0	Possibly damaging (0.838)	Polymorphism		Heterozygous	CM076486
10	29870	Retinitis pigmentosa	*BBS9*	NM_198428.2:c.2363C>T:p.Ser788Phe	Deleterious	Bad	C15	Probably damaging (0.997)	Disease-causing		Heterozygous	This study
			CHM	NM_000390.2:c.223T>C:p.Trp75Arg	Deleterious	Bad	C0	Probably damaging (0.994)	Disease-causing		Heterozygous	This study
11	71703	Cone-rod dystrophy	*C2orf71*	NM_014704.3:c.1966C>T:p.Arg656Cys	Deleterious	Bad	C0	Probably damaging (1)	Disease-causing		Heterozygous	This study
			COL11A2	NM_080680.2:c.388C>T:p.Arg130Trp	Deleterious	Bad	C0	Probably damaging (1)	Disease-causing		Heterozygous	This study
			PEX1	NM_000466.2:c.3208-1G>A						Splice acceptor lost	Heterozygous	This study
			FZD4	NM_012193.3:c.356G>T:p.Gly119Val	Deleterious	Bad	C15	Benign (0.014)	Disease-causing		Heterozygous	This study
			RPGRIP1L	NM_015272.3:c.1340T>C:p.Leu447Ser	Tolerated		C25	Benign (0.01)			Heterozygous	CM093292
			AIPL1	NM_014336.4:c.244C>T:p.His82Tyr	Tolerated		C0	Possibly damaging	Polymorphism		Heterozygous	CM034202

								(0.666)				
12	71918	Retinitis pigmentosa	*PDE6B*	NM_000283.3:c.794G>A:p.Arg265Gln	Deleterious	Bad	C0	Probably damaging (1)	Disease-causing	New acceptor site	Heterozygous	This study
			PROM1	NM_006017.2:c.1142-1G>A						New acceptor site	Heterozygous	CS107464
			VCAN	NM_004385.4:c.2234A>C:p.Lys745Thr	Deleterious	Bad	C0	Probably damaging (0.958)	Polymorphism		Heterozygous	This study
			ITGB3	NM_000212.2:c.1576G>C:p.Glu526Gln	Deleterious	Bad	C0	Probably damaging (0.981)	Disease-causing		Heterozygous	This study
			CACNA1F	NM_005183.3:c.4118T>C:p.Val1373Ala	Deleterious		C25	Probably damaging (0.948)	Disease-causing		Heterozygous	This study
13	71471	Retinitis pigmentosa	*ABCA4*	NM_000350.2:c.3113C>T:p.Ala1038Val	Deleterious	Bad	C65	Benign (0.009)	Disease-causing		Heterozygous	CM970006
			CRB1	NM_201253.2:c.2842T>C:p.Cys948Arg	Deleterious	Bad	C66	Probably damaging (0.998)	Disease-causing	Cryptic Donor Strongly Activated	Heterozygous	This study
			GRM6	NM_000843.3:c.1162C>T:p.Arg388Cys	Deleterious	Bad	C0	Probably damaging (1)	Disease-causing		Heterozygous	This study
14	71728	Retinitis pigmentosa	*ADGRV1*	NM_032119.3:c.17017A>G:p.Lys5673Glu	Deleterious	Bad	C0	Possibly damaging (0.748)	Disease-causing		Heterozygous	This study
15	28865	Retinitis pigmentosa	*IMPG2*	NM_016247.3:c.3038C>T:p.Pro1013Leu	Deleterious	Bad	C65	Probably damaging (0.976)	Disease-causing		Heterozygous	This study
			TRIM32	NM_001099679.1:c.314G>T:p.Arg105Leu	Deleterious	Bad	C15	Possibly damaging (0.701)	Disease-causing		Heterozygous	This study
16	24058	Retinitis pigmentosa	*PDE6B*	NM_000283.3:c.1059+51G>A						New acceptor site	Homozygous	This study
			BBS7	NM_176824.2:c.36+20C>T						New donor site	Heterozygous	This study

			DHX38	NM_014003.3:c.901 G>A:p.Glu301Lys	Deleterious	Bad	C55	Benign (0.026)	Disease-causing		Heterozygous	This study
17	71192	Retinal dystrophy	*HMCN1*	NM_031935.2:c.8815G>A:p.Gly2939Ser	Deleterious	Good	C55	Probably damaging (0.987)	Disease-causing		Heterozygous	This study
18	71762	Retinitis pigmentosa	*IMPG2*	NM_016247.3:c.283 G>A:p.Glu95Lys	Deleterious	Bad	C15	Probably damaging (0.986)	Disease-causing		Heterozygous	This study
			ADGRV1	NM_032119.3:c.2834G>A:p.Gly945Glu	Deleterious	Bad	C0	Probably damaging (0.998)	Disease-causing		Heterozygous	This study
			BBS9	NM_198428.2:c.1967G>A:p.Arg656Gln	Deleterious	Bad	C0	Probably damaging (1)	Disease-causing		Heterozygous	This study
			TRIM32	NM_001099679.1:c.558G>C:p.Gln186His	Deleterious	Bad	C15	Probably damaging (0.948)	Disease-causing		Heterozygous	This study
			ZNF408	NM_024741.2:c.1342C>T:p.Arg448Cys	Deleterious	Bad	C65	Probably damaging (0.993)	Disease-causing		Heterozygous	This study
			GNPTG	NM_032520.4:c.502 G>A:p.Val168Ile	Deleterious	Bad	C25	Probably damaging (0.999)	Disease-causing		Heterozygous	This study
			PDE6G	NM_002602.3:c.136 G>C:p.Gly46Arg	Deleterious	Bad	C65	Probably damaging (1)	Disease-causing		Heterozygous	This study
19	72007	Retinitis pigmentosa	*CNGA3*	NM_001298.2:c.847 C>T:p.Arg283Trp	Deleterious	Bad	C65	Probably damaging (1)	Disease-causing		Heterozygous	CM980376
			ARL6	NM_001278293.1:c.349+36G>A						New acceptor site	Heterozygous	This study
			NPHP3	NM_153240.4:c.1189C>T:p.Arg397Cys	Tolerated	Bad	C0	Probably damaging (0.992)	Disease-causing		Heterozygous	CM077306
			RP1	NM_006269.1:c.615 +3G>A						Cryptic Donor Strongly Activated	Heterozygous	This study

			PHYH	NM_006214.3:c.734G>A:p.Arg245Gln	Deleterious	Bad	C0	Probably damaging (0.948)	Disease-causing		Heterozygous	CM001299
			BBS10	NM_024685.3:c.1631A>G:p.Asn544Ser	Tolerated	good	C0	Benign (0)	Polymorphism		Heterozygous	CM1010462
			MKKS	NM_018848.3:c.1553G>A:p.Arg518His	Tolerated	good	C1	Benign (0)	Polymorphism		Heterozygous	CM010926
20	13730	Retinitis pigmentosa	*ABCA4*	NM_000350.2:c.1302del:p.Gln437Argfs*12							Heterozygous	CD156176
			NBAS	NM_015909.3:c.6466dup:p.Arg2156Profs*12							Heterozygous	This study
			CNGB1	NM_001297.4:c.2747G>A:p.Arg916His	Deleterious	Bad	C25	Probably damaging (1)	Disease-causing		Heterozygous	CM119879
21	30421	Leber Congenital Amaurosis	*ABCA4*	NM_000350.2:c.688T>A:p.Cys230Ser	Deleterious	Bad	C65	Probably damaging (0.992)	Disease-causing		Heterozygous	CM003361
			HMCN1	NM_031935.2:c.1535C>T:p.Thr512Ile	Deleterious	Bad	C0	Probably damaging (0.995)	Disease-causing		Heterozygous	This study
			CRB1	NM_201253.2:c.4039del:p.Thr1347Leufs*5							Heterozygous	This study
			NBAS	NM_015909.3:c.5465A>C:p.Asn1822Thr	Deleterious		C55	Probably damaging (1)	Disease-causing		Heterozygous	This study
22	71315	Retinitis pigmentosa	*IMPG2*	NM_016247.3:c.1300C>T:p.Pro434Ser	Deleterious	Bad	C0	Probably damaging (0.993)	Disease-causing		Heterozygous	This study
			BBS12	NM_152618.2:c.116T>C:p.Ile39Thr	Deleterious	Bad	C1	Probably damaging (0.988)	Disease-causing		Heterozygous	CM1010465
			COL9A1	NM_001851.4:c.2159G>A:p.Arg720Gln	Deleterious	Bad	C35	Probably damaging (1)	Disease-causing		Heterozygous	This study
			CDH23	NM_022124.5:c.2714C>T:p.Ala905Val	Deleterious	Bad	C65	Benign (0.112)	Disease-causing	New donor site	Heterozygous	This study

			RPGRIP1	NM_020366.3:c.1767G>T:p.Gln589His	Deleterious	Bad	C0	Probably damaging (0.995)	Disease-causing		Heterozygous	CM057749
23	29303	Retinitis pigmentosa	*DTHD1*	NM_001170700.2:c.1442T>C:p.Leu481Pro	Deleterious		C25	Probably damaging (0.994)	Disease-causing		Heterozygous	This study
			CDH23	NM_022124.5:c.1307G>A:p.Ser436Asn	Deleterious	Bad	C45	Probably damaging (0.991)	Disease-causing		Heterozygous	This study
			ROM1	NM_000327.3:c.686G>A:p.Arg229His	Tolerated	Good	C0	Benign (0.048)	Polymorphism		Heterozygous	CM104430
			BBS1	NM_024649.4:c.24T>C:p.Asp8Asp							Heterozygous	CM085270
24	71133	Leber Congenital Amaurosis	*IFT172*	NM_015662.2:c.4611C>G:p.Ile1537Met	Deleterious	Not scored	C0	Probably damaging (0.96)	Not scored		Homozygous	This study
			ALMS1	ENST00000264448.6:c.8237T>G:p.Val2746Gly	Deleterious		C0	Probably damaging (0.899)	Disease-causing		Heterozygous	This study
			CNGB3	NM_019098.4:c.2420C>G:p.Ala807Gly	Tolerated	Good	C0	Benign (0.437)	Polymorphism	New donor site	Heterozygous	This study
			PHYH	NM_006214.3:c.734G>A:p.Arg245Gln	Deleterious	Bad	C0	Probably damaging (0.948)	Disease-causing		Heterozygous	CM001299
			BEST1	NM_001139443.1:c.857C>A:p.Pro286His	Deleterious	Bad	C0	Probably damaging (1)	Disease-causing		Heterozygous	CM1511254
			FZD4	NM_012193.3:c.118G>C:p.Glu40Gln	Deleterious	Bad	C25	Benign (0.389)	Disease-causing		Heterozygous	CM103438
25	71161	Leber Congenital Amaurosis	*USH2A*	NM_206933.2:c.15433G>A:p.Val5145Ile	Deleterious	Bad	C0	Possibly damaging (0.553)	Disease-causing		Heterozygous	CM140609
			VPS13B	NM_017890.4:c.4157+5A>G						Cryptic Donor Strongly Activated	Heterozygous	This study
			RPGRIP1	NM_020366.3:c.2017C>T:p.Gln673*							Heterozygous	This study
			BBS10	NM_024685.3:c.163	Tolerated	Good	C0	Benign (0)	Polymorp		Heterozygo	CM101046

				1A>G:p.Asn544Ser					hism		us	2
			MKS1	NM_017777.3:c.857 A>G:p.Asp286Gly	Deleterious	Bad	C0	Possibly damaginig (0.949)	Disease-causing		Heterozygous	CM081333
26	70946	Best macular dystrophy	*USH2A*	NM_206933.2:c.6240G>T:p.Lys2080Asn	Tolerated	Good	C0	Possibly damaginig (0.883)	Polymorphism		Heterozygous	CM155319
			GRM6	NM_000843.3:c.1292C>T:p.Pro431Leu	Tolerated	Bad	C0	Probably damaging (0.982)	Disease-causing		Heterozygous	This study
			INPP5E	NM_019892.4:c.746 C>T:p.Ser249Phe	Deleterious		C0	Probably damaging (0.999)	Disease-causing		Heterozygous	This study
27	70559	Best macular dystrophy	*CCDC40*	NM_017950.2:c.2251C>A:p.Pro751Thr	Deleterious	Bad	C0	Probably damaging (0.998)	Disease-causing		Heterozygous	This study
			CEP164	NM_014956.4:c.190 C>G:p.Pro64Ala	Deleterious	Bad	C25	Possibly damaging (0.471)	Disease-causing		Heterozygous	This study
			GRM6	NM_000843.3:c.1732C>T:p.Arg578Cys	Deleterious	Bad	C15	Possibly damaging (0.629)	Disease-causing		Heterozygous	This study
			IMPG2	NM_016247.3:c.263 G>T:p.Gly88Val	Deleterious	Bad	C65	Probably damaging (1)	Disease-causing	Cryptic Acceptor Strongly Activated	Heterozygous	This study
			MKKS	NM_170784.1:c.1015A>G:p.Ile339Val	Tolerated	Good	C0	Benign (0.008)	Polymorphism	New donor site	Heterozygous	CM021299
			VPS13B	NM_017890.4:c.3386A>G:p.Lys1129Arg	Tolerated	Good	C0	Benign (0.125)	Disease-causing	New donor site	Heterozygous	This study
			PCDH15	NM_001142763.1:c.*14916G>A						New acceptor site	Heterozygous	This study
			KIF7	NM_198525.2:c.1015A>C:p.Thr339Pro	Deleterious	Unknown	C0	Probably damaging (0.977)	Disease-causing		Heterozygous	This study
			ITGB3	NM_000212.2:c.785 T>C:p.Ile262Thr	Deleterious	Bad	C65	Probably damaging (0.998)	Disease-causing		Heterozygous	This study

28	71583	Retinal dystrophy	*LRP5*	NM_002335.2:c.2489C>T:p.Ser830Leu	Deleterious	Bad	C65	Probably damaging (1)	Disease-causing		Heterozygous	This study
			PCYT1A	NM_001312673.1:c.217+5G>A							Heterozygous	This study
29	71749	Retinitis pigmentosa	*CDHR1*	NM_033100.3:c.783G>A:p.Pro261Pro							Heterozygous	CS140565
			EMC1	NM_015047.2:c.2641C>T:p.Arg881Cys	Deleterious	Bad	C25	Probably damaging (1)	Disease-causing		Heterozygous	This study
			LCA5	NM_001122769.2:c.661T>G:p.Leu221Val	Deleterious	Bad	C0	Probably damaging (1)	Disease-causing		Heterozygous	This study
30	30806	Bardet-Biedl Syndrome	*CDH23*	NM_022124.5:c.3845A>G:p.Asn1282Ser	Deleterious	Bad	C45	Possibly damaging (0.933)	Disease-causing		Heterozygous	This study
			DHX29	NM_019030.2:c.3670C>A:p.Leu1224Met	Deleterious	Bad	C0	Probably damaging (0.998)	Disease-causing		Heterozygous	This study
			KIAA0556	NM_015202.2:c.27dup:p.Glu10Argfs*16							Heterozygous	This study
31	27419	Usher syndrome	*DNAH5*	NM_001369.2:c.8757G>C:p.Glu2919Asp	Deleterious	Bad	C0	Benign (0.190)	Disease-causing		Heterozygous	This study
			PARVA	NM_018222.4:c.425T>C:p.Ile142Thr	Deleterious	Bad	C0	Possibly damaging (0.739)	Disease-causing		Heterozygous	This study
			PDE6A	NM_000440.2:c.1926+1G>A:p.?						Donor site lost	Heterozygous	This study
			ROM1	NM_000327.3:c.47G>A:p.Arg16His	Deleterious	Bad	C0	Possibly damaging (0.82)	Polymorphism		Heterozygous	This study
			TRPM1	NM_001252020.1:c.511G>A:p.Gly171Arg	Deleterious	Bad	C65	Probably damaging (1)	Disease-causing		Heterozygous	This study
32	25939	Retinitis pigmentosa	*ABCA4*	NM_000350.2:c.466A>G:p.Ile156Val	Tolerated	Good	C0	Benign (0.002)	Disease-causing		Heterozygous	CM003569
			CDHR1	NM_033100.3:c.1367C>A:p.Ala456Glu	Deleterious	Unknown	C0	Probably damaging (1)	Disease-causing		Heterozygous	This study

			DHX38	NM_014003.3:c.2838G>T:p.Met946Ile	Deleterious	Bad	C0	Probably damaging (0.995)	Disease-causing		Heterozygous	This study
			FAM161A	NM_001201543.1:c.881C>T:p.Pro294Leu	Deleterious	Bad	C65	Probably damaging (1)	Disease-causing		Heterozygous	This study
			MKKS	NM_018848.3:c.1015A>G:p.Ile339Val	Tolerated	Good	C0	Benign (0.008)	Polymorphism	New donor site	Heterozygous	CM021299
			NPHP4	NM_015102.4:c.3364A>C:p.Thr1122Pro	Tolerated	Unknown	C0	Probably damaging (0.967)	Unknown		Heterozygous	CM110621
			USH2A	NM_206933.2:c.12343C>T:p.Arg4115Cys	Deleterious	Bad	C65	Benign (0.167)	Disease-causing		Heterozygous	CM045641
33	71868	Retinitis pigmentosa DD: Retinal dystrophy	*IMPG2*	NM_016247.3:c.926A>G:p.Tyr309Cys	Deleterious	Bad	C55	Probably damaging (1)	Disease-causing		Heterozygous	This study
34	71808	Retinitis pigmentosa	*COL11A1*	NM_080629.2:c.4452C>A:p.Asp1484Glu	Deleterious	Bad	C35	Probably damaging (0.996)	Disease-causing		Heterozygous	This study
			COL9A1	NM_001851.4:c.1349A>G:p.Glu450Gly	Deleterious	Bad	C65	Probably damaging (1)	Disease-causing		Heterozygous	This study
			IFT172	NM_015662.2:c.4793A>G:p.Asn1598Ser	Deleterious	Unknown	C0	Probably damaging (1)	Unknown	New acceptor site	Heterozygous	This study
			MKS1	NM_017777.3:c.1388G>A:p.Arg463Gln	Tolerated	Good	C0	Benign (0.175)	Disease-causing	New acceptor site	Heterozygous	This study
			RGR	NM_002921.3:c.505G>T:p.Asp169Tyr	Deleterious	Bad	C65	Probably damaging (1)	Disease-causing	Cryptic acceptor strongly activated	Heterozygous	This study
			USH2A	NM_206933.2:c.10931C>T:p.Thr3644Met	Deleterious	Bad	C65	Probably damaging (0.966)	Disease-causing		Heterozygous	CM149213
35	71094	Cone-rod dystrophy	*CRB1*	NM_201253.2:c.135C>G:p.Cys45Trp	Deleterious	Bad	C65	Probably damaging	Disease-causing		Heterozygous	CM107848

								(1)				
			LGR4	NM_018490.2:c.2359C>G:p.Leu787Val	Deleterious	Bad	C25	Probably damaging (0.999)	Disease-causing		Heterozygous	This study
			MKKS	NM_018848.3:c.724G>T:p.Ala242Ser	Deleterious	Good	C15	Probably damaging (0.987)	Disease-causing		Heterozygous	CM001242
			USH2A	NM_206933.2:c.6632G>T:p.Gly2211Val	Deleterious	Bad	C65	Probably damaging (1)	Disease-causing		Heterozygous	This study
36	71718	Stargardt disease	*ABCA4*	NM_000350.2:c.5882G>A:p.Gly1961Glu	Deleterious	Bad	C65	Probably damaging (1)	Disease-causing		Heterozygous	CM970016
			PDE6B	NM_000283.3:c.655T>C:p.Tyr219His	Tolerated	Good	C0	Benign (0.025)	Polymorphism		Heterozygous	CM942034
37	71780	Macular dystrophy	*CNNM4*	NM_020184.3:c.973G>A:p.Asp325Asn	Deleterious	Bad	C15	Probably damaging (0.999)	Disease-causing		Heterozygous	This study
			DHX38	NM_014003.3:c.1379G>A:p.Arg460His	Deleterious	Bad	C25	Probably damaging (1)	Disease-causing		Heterozygous	This study
			DHX38	NM_014003.3:c.3098T>C:p.Ile1033Thr	Deleterious	Bad	C65	Probably damaging (0.993)	Disease-causing		Heterozygous	This study
			RAX2	NM_032753.3:c.92G>A:p.Arg31His	Deleterious	Bad	C25	Probably damaging (1)	Unknown		Heterozygous	This study
			RP1L1	NM_178857.5:c.1138G>A:p.Gly380Arg	Deleterious	Bad	C65	Probably damaging (0.959)	Disease-causing		Heterozygous	This study
